# Finding Opportunities for Health-Care System Advancement in the COVID-19 Crisis

**DOI:** 10.1017/dmp.2020.402

**Published:** 2020-10-22

**Authors:** Richard G. Malish, Garrett Meyers, Charles D. Sondgeroth, Michael S. Whiddon, Brian T. Hall

**Affiliations:** Darnall Army Medical Center, Fort Hood, Texas, USA

**Keywords:** health care economics and organizations, military medicine, crises planning

## Abstract

The coronavirus disease 2019 (COVID-19) pandemic forced American medical systems to adapt to high patient loads of respiratory disease. Its disruption of normal routines also brought opportunities for broader reform. The purpose of this article is to describe how the Carl R. Darnall Army Medical Center (CRDAMC), a medium-sized Army hospital, capitalized on opportunities to advance its strategic aims during the pandemic. Specifically, the hospital sequentially adopted virtual video visits, surged on preventative screenings, and made-over its image to appeal to patients seeking urgent care. These campaigns supported COVID-19 efforts and larger strategic goals simultaneously, and they will endure for years to come. Predictably, CRDAMC encountered obstacles in the course of its transformation. These obstacles and their follow-on lessons are provided to assist future medical leaders seeking quantum change in the opportunities made available by health crises.

The Centers for Disease Control and Prevention (CDC) divides the pandemic continuum into interpandemic, alert, pandemic (response), and transition (recovery) phases.^1^ If well resourced, a system can rebuild itself into a more stable and modern version of its precrisis self during the transition phase. This article describes how a military hospital rapidly achieved systemic improvements during the pandemic and transition phases of the coronavirus disease 2019 (COVID-19) crisis. Spared from significant inpatient burdens, the hospital seized opportunities provided by the pandemic. In doing so, it emerged from the crisis far better suited, as compared to its baseline, to achieve its goals.

Carl R. Darnall Army Medical Center (CRDAMC) is one of the US Army’s 8 Medical Centers. It is the tertiary referral center for 12 primary care clinics located in Fort Hood, Texas, and the civilian communities surrounding the post. Of the almost 100,000 patients enrolled in the system, approximately 37,000 are soldiers, 30,000 are family members (including children), and the remainder are retired soldiers and their families. The Hospital’s Incident Command System (HICS) is similar in size and composition to that of civilian counterparts.^2^ Additionally, the leadership team has combat experience and skillsets acquired from broad professional military education. These features create a crew comfortable in stressful conditions and adept at exploiting advantages in dynamic milieus. Finally, CRDAMC is funded by tax-payer dollars. The reliability of this capital stream provides freedoms not available to most civilian hospitals who must treat as many patients as possible to generate revenue.

In March 2020, as COVID-19 erupted in the United States, CRDAMC leaders established respiratory illness wards and clinics, acquired testing materials, rerouted hospital workflows, and monitored its inventory of protective equipment. In mid-March, at the direction of the State of Texas and the US Army, the hospital postponed all elective surgeries and routine care.^3,4^ In following jurisdictional guidance, CRDAMC’s COVID-19 posture generally resembled that of nearby civilian hospitals and distant military ones.

For reasons that may never be known, but perhaps because of its young demographic, dispersed population, and successful implementation of public health measures, CRDAMC was spared the COVID-19 devastation seen elsewhere. Due to its preemptive restrictions on routine care and elective procedures, CRDAMC’s clinics and wards instead became substantially empty. This development allowed hospital staff to prioritize other tasks. In May 2020, the hospital changed its stance from a focus exclusively on COVID-19 to one that included both continuous preparedness for COVID-19 and the re-ignition of the health-care system that had previously dutifully maintained the health of the population. It is CRDAMC’s approach to the latter effort that is the focus of this article. Instead of simply resuming operations to refill its vacated capacity, CRDAMC moved out deliberately and asymmetrically, signaling new priorities and seizing opportunities made available by the crisis. In doing so, it made advances toward its strategic aims.

## Methods

CRDAMC separated its return to full operations into 3 phases, focusing on a specific campaign objective in each phase.

First, to remain available to patients despite COVID-19 care restrictions, CRDAMC converted its primary care approach to one that emphasized contact through virtual mechanisms. Specifically, CRDAMC leaders acquired Virtual Video Visit (V3) software and hardware and introduced the technology to all 12 primary clinics simultaneously.^5^ This approach represented a change to a previously published plan to deploy V3 technology sequentially to each clinic over years. Leaders rapidly trained administrative staff, clinical staff, and patients on its use. After converting appropriate face-to-face (F2F) appointments to virtual ones, CRDAMC embarked toward a paradigm of care that it had only begun to test before the pandemic. In doing so, it exponentially shortened pre-COVID-19 timelines for the transition.

Second, influenced by data demonstrating national declines in the use of emergency and urgent care,^6^ and sensitized to the pragmatic effects that avoidance of routine care might have on chronically ill and at-risk beneficiaries, CRDAMC reached out to its sickest patients. The goal was to ensure that they had the health-care resources needed to prevent a “second wave” of morbidity and mortality associated with reduced access to F2F care.^7,8^ Using similar logic, the hospital surged in its outreach related to cancer screening, vaccinations, and management of diabetes to avoid mid- to long-term future waves of morbidity and mortality due to lapses in prevention. Improvement, not merely sustainment, was the objective. Leaders directed clinicians to use the time made available by COVID-19 restrictions to eliminate a preexisting backlog of screening studies.

In the third phase, the system re-designated its 12 primary care sites as urgent care centers, creating continuous real-time access with the capacity liberated by converting F2F appointments to shorter virtual visits. Unlike other military hospitals, CRDAMC kept all of its clinics open to minimize disruptions of routines for patients and staff. To make sure outlets were prepared for urgent care in the midst of a pandemic, leadership equipped all outlying primary care clinics with COVID-19 personal protective equipment, screening personnel, and testing resources. Public affairs teammates ensured that patients understood that their clinics were open and available for urgent visits. They did so by orchestrating a media blitz that included notices placed in the hospital’s closed-circuit television rotation, posts on social media, verbal announcements in community meetings, and articles in the local press.^9^ The goal of this approach was to create unobstructed access so that patients (sick with COVID-19 or otherwise) could quickly and easily be seen.

In all aspects of change, CRDAMC rebuilt itself with endurance in mind, concerned that COVID-19 facilitated reform might regress to the status quo when the crisis diminished.

## Results

**Figure 1.**
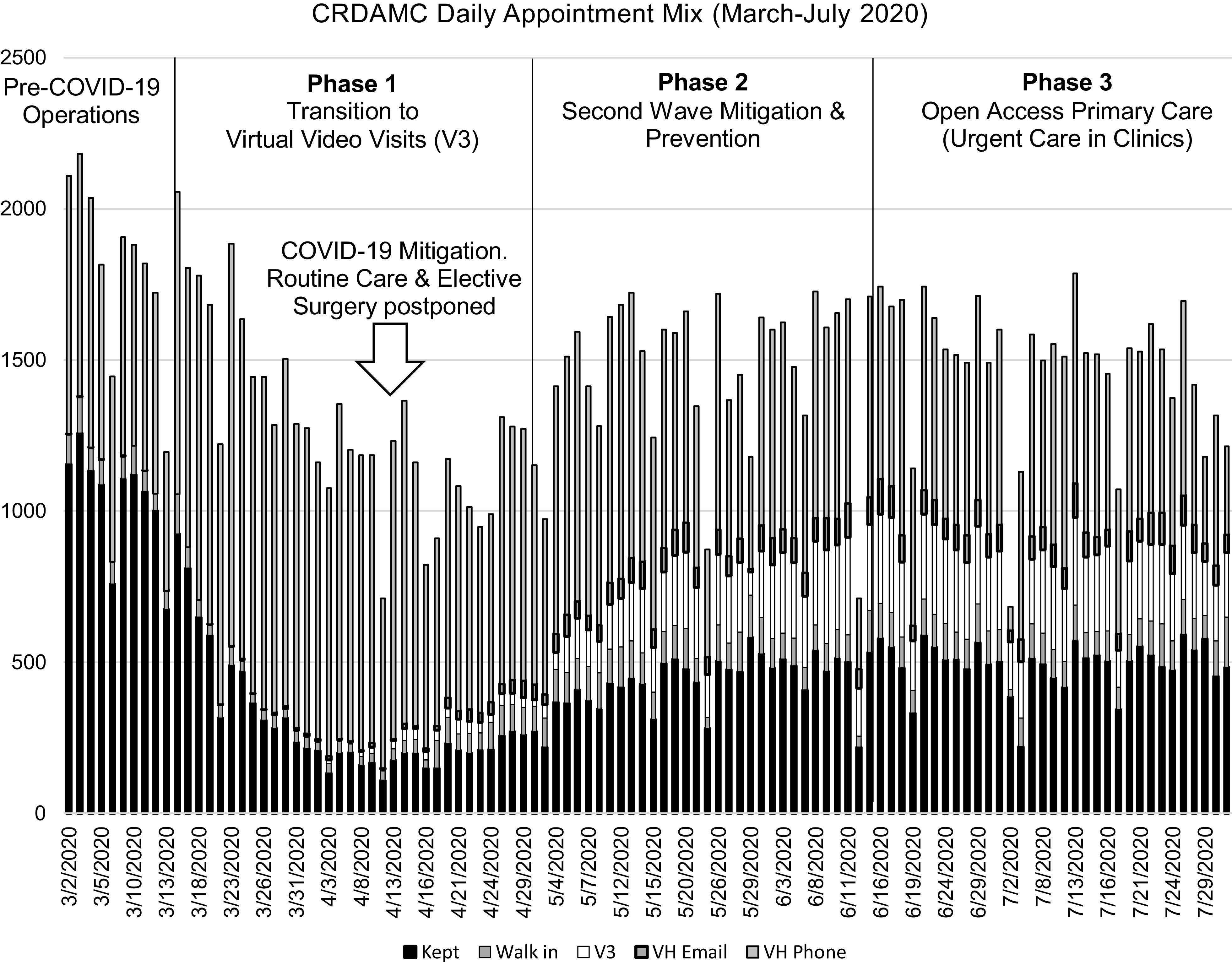
The mix of appointment types during Pre-COVID-19 operations through CRDAMC’s 3 phases of COVID-19 mitigation. Of note, V3s came into existence and quickly grew to become a significant source of healthcare delivery. Kept (F2F) appointments increased during Phase 2 as primary care outlets surged on preventative screenings and second wave mitigation. Walk-ins are becoming more frequent in Phase 3, but additional time is needed to see long-lasting behavioral changes.

Figure 1 demonstrates the impacts of each phase on primary care encounter types. In early March 2020, CRDAMC conducted none of its care through V3s. Four months into the COVID-19 pandemic, CRDAMC averaged 250 V3s per day. The health-care system introduced V3s and rapidly propagated them to account for 25-30% of all patient contacts. Notably, the primary care team transformed from one in which the majority of its patient contacts were F2F (approximately 1200 per day) to one in which the majority of contacts are made through telephone calls, email, and V3s. For context, from March 20 to July 20, despite its “medium” size, CRDAMC became the #1 user of virtual video technology in the Department of Defense.^5^ Its use of V3s was 2.5 times more than its nearest competitor (25,000 vs 10,000 encounters).

In the second campaign, public health nursing analysts queried the military’s electronic medical record to identify CRDAMC outpatients with 8 high-morbidity Expanded Diagnosis Clusters (EDCs; asthma, anxiety, cancer, cardiovascular disease, chronic obstructive pulmonary disease, congestive heart failure, depression, diabetes) who had not accessed care in the past 120 days. Nurse case managers called each, identifying which patients required appointments, refills, specialty referrals, and/or other care. In 6 weeks, CRDAMC clinical staff called its 2000 sickest patients and coordinated care for 264 (13.2%). Such outreach will continue indefinitely.

**Figure 2.**
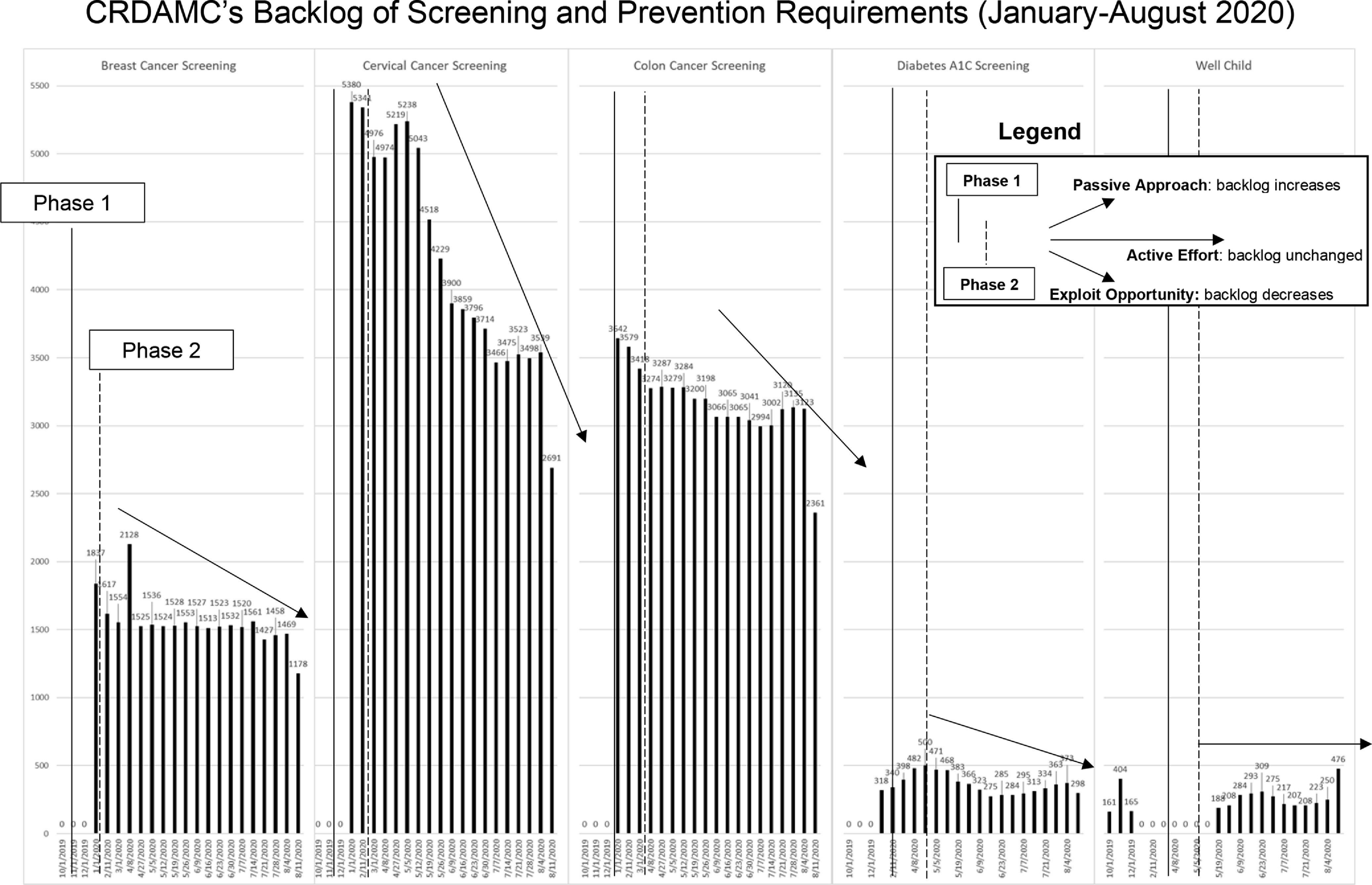
The predicted increase in backlog of screening and prevention studies as CRDAMC reduced elective and routine care (Phase 1). Its Phase 2 efforts, however, did more than maintain the existing backlog. CRDAMC reduced the backlog (in 4 of 5 measures) by using the time made available from the reduced demand for nonpreventative routine care.

Furthermore, CRDAMC used its increased capacity to decrease its preexisting backlog of routine screening exams. In 4 months, CRDAMC performed over 4000 studies. Preventative visits (cervical exams and F2F care for high-morbidity EDC patients) account for the uptick in F2F visits seen in Phase 2 of Figure 1. The absolute decrease in overdue screening exams is demonstrated in Figure 2. Were it not for emphasis on second wave mitigation, one would expect the screening backlog to grow rather than shrink in a period in which routine care was dis-incentivized. Such will undoubtedly be the pattern nationally. The United States witnessed declines of 86-94% in routine cancer screens during the middle portion of 2020.^10^ CRDAMC also maintained its A1C and well-child screening/vaccination backlogs during COVID-19. More importantly, it advanced its HEDIS (Healthcare Effectiveness Data and Information Set) metrics from the national average to the top 25% in breast cancer and the top 10% in cervical cancer screening. Furthermore, CRDAMC advanced from the top 25% to top 10% in colon cancer screening. While prevention may be considered routine business, a forward-leaning, team-based outlook allowed a potential period of regression to become a surge forward.

**Figure 3.**
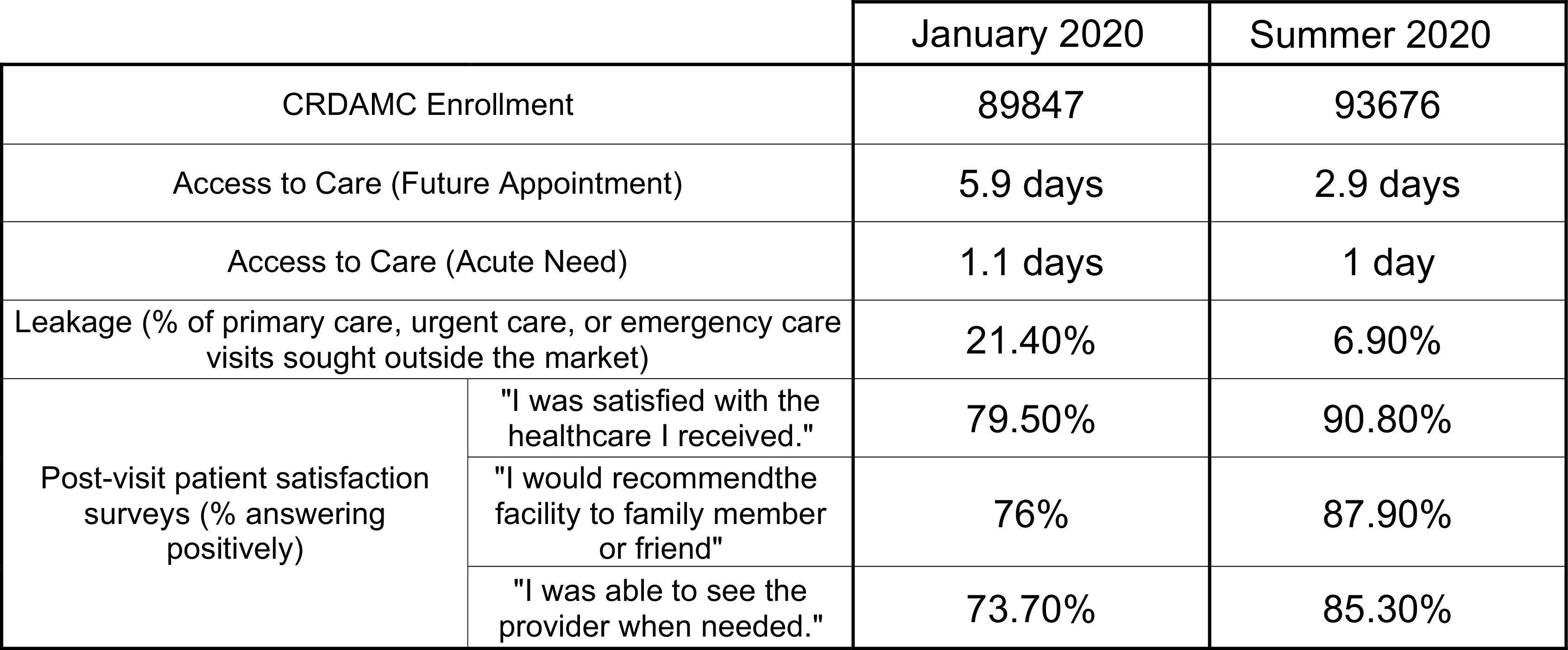
The impact of CRDAMC’s transformation to an urgent care methodology.

As depicted in Figure 3, the campaign to improve access to urgent care was successful. It reduced the amount of patients seeking care outside of the CRDAMC system (leakage) and contributed to markedly improved patient satisfaction scores in postvisit surveys.

The 3 campaigns were thematically linked in that they were consistent with the behaviors incentivized by a value-based health-care system as opposed to those of a fee-for-service (FFS) -based model. Capitation is the method of reimbursement most often used to achieve value. In capitated models, the managed care organization (payer) remits a fixed payment at regular intervals for each enrolled patient regardless of the number or nature of services rendered.^11^ This model discourages unnecessary care and encourages positive outcomes (good health, cancer prevention, excellent patient experience, and rare use of the health-care system). In FFS models, conversely, hospitals accumulate revenue by billing insurance companies for each episode of care. The FFS model encourages high productivity and, thus, frequent in-person visits, tests, procedures, and surgeries. Patient satisfaction, healthy outcomes, and convenience are not directly rewarded.

Because the Army is tax-payer funded, it uses reimbursement models, not for payment, but to influence provider behavior and to guide outcomes. Within the past decade, the Army adopted a framework similar to the Institute of Healthcare Improvement’s Triple Aim (Better Care, Better Health, Lower Cost),^12^ signaling a commitment to value. To achieve value-based goals, it transitioned from a FFS model to a capitated reimbursement model for primary care in 2013. Even so, the system has not been able to completely transmit capitation-driven action from the strategic level to the operational level. Before COVID-19, individual clinics continued to conduct operations on a backbone of policy and templating designed for maximal productivity. Reinventing its primary care clinics as QUiC (Quality, Urgent, internet and phone Care) clinics, CRDAMC used the opportunities offered in the pandemic to eliminate low-health-value work that would only be performed in FFS models.^13^ The benefits of patient convenience (attained by virtual outreach), urgent care access, and prevention are more “in tune” with a value-based health-care system offering both medical services and medical insurance.

## Discussion

The strategic transformation due to COVID-19 improved the overall efficiency and effectiveness of CRDAMC compared with its precrisis baseline. Even so, the transformation was neither simple nor free of friction. CRDAMC learned lessons along the way. The following lessons may be of value to hospitals seeking similar reform in the opportunity-rich environment of a future health-care crisis.

### Lesson 1: Health-Care Systems Should Adjust Their Emergency Response to Match Local Conditions Rather Than National Predictions

Directed to do so by the US Army and the State of Texas, CRDAMC’s abrupt halting of routine and elective care was unavoidable. Leaders at the State and Army level made the decision to “conserve personal protective equipment (PPE) and free up staff and facilities for COVID-19 patients.”^4^ The decision reveals the widespread supposition that the predicted risk of COVID-19 was greater than the accepted risk of depriving chronically ill patients of real-time care and well patients of preventative screening. Whether this hypothesis is correct remains to be seen.

State and enterprise directives cannot be ignored. Even so, individual hospitals have significant latitude in their implementation. Both directives, in fact, specified that their prescribed action should be customized based on local considerations. Unlike other military hospitals, CRDAMC kept all facilities and wards open and staffed even while reducing services. This nuanced approach was not universally popular at CRDAMC. Even so, it created, for leaders, the opportunity to pursue the 3-phased reopening while retaining the flexibility to pivot back to a pure COVID-19 response if needed.

According to Klein and Irizarry, “a vital core concept within the field of disaster management is that all disasters are local.”^14^ If so, risk-based decisions are best approached along a continuous scale—informed by local conditions, rather than a dichotomous one—informed by regional concerns. State declarations and country-wide projections are too imprecise to be used singularly and effectively at the local level. CRDAMC soon learned to weigh local variables more heavily in its risk-benefit decisions. Among such variables was a high sensitivity to the impacts of denying services to the chronically ill. As subsequent waves of COVID-19 developed, CRDAMC either dialed up or down its posture, but never again eliminated “routine” services to those in need.

### Lesson 2: Ceasing Routine Operations Creates the Time, Opportunities, Focus, and Capacity to Launch New Campaigns

Although characterized by the downsides mentioned, the opportunities made available by powering down a continuously operating hospital cannot be overstated. The accelerated action achieved by the phased reopening could only have emanated from a stationary and recoiled position. CRDAMC’s phased reopening fostered undivided whole-of-hospital attention on the 3 campaigns in sequence. The result was quantum, rather than incremental, advancement in each. While such progress is theoretically possible in routine times, it is difficult in practice due to a lack of capacity, unity of effort, and enthusiasm for change.

Of interest, the strategic reopening pursued by CRDAMC was not reproduced by civilian neighbors because of their need for revenue. When safety was assured, local partners turned their systems back “on” en masse, with new COVID-19 mitigation in place. As such, they were unable to duplicate the performance improvement accomplished by CRDAMC.

The military uses the term “stand down” to describe deliberate pauses in operations to address focused areas. Military officers use stand downs almost exclusively to emphasize safety procedures in the aftermath of accidents or other tragedies.^15^ The CRDAMC COVID-19 experience makes the case for the use of proactive, planned, full-scale stand downs to catalyze major strategic change. Decreasing hospital services to focus staff on strategic goals can be powerful if used wisely.

### Lesson 3: Changing the Status Quo Requires Sustained Attention

Early in the COVID-19 response, the term “the new normal” became a popular watchword. Scholars and pundits accepted that the post-COVID paradigm would have both positive and negative features. That positive aspects of the new normal would flow irrefutably from the current normal, however, was an incorrect assumption. CRDAMC’s first probing attempts at change quickly regressed to baseline conditions. Such experience led the leadership team to understand that the status quo has advantages that can defeat even crisis-driven revolutions. If habitual behaviors can outlast the initial urgency for change, organizational enthusiasm may dissipate and, along with it, the promise of a new normal.

Recognizing that the COVID-19-generated excess capacity was accompanied by a fleeting window of time to achieve forward movement, leaders condensed typical timelines for action, relying on tempo and resolve to overcome inertia. CRDAMC leaders maintained momentum by meeting daily to focus on measures of effectiveness of new workflows. In this manner, CRDAMC made sure that COVID-19 opportunities did not pass without exploitation.

### Lesson 4: Campaign Plans Are Expeditiously Executed, not Created, in Times of Turmoil

Strategic planning typically develops campaigns to correct the inadequacies of a system. Such inadequacies are magnified or highlighted (and rarely discovered) when crises arise. As a result, solving acute crisis-driven challenges should have synergistic effects with the organization’s strategic direction, if that direction is well-known and well-published. CRDAMC quickly pivoted to the adjustments mandated by COVID-19 because the organization was aware, before the crisis struck, that they needed to be made. *Of all of the changes CRDAMC made in the wake of COVID-19, none required a pandemic to reveal them as needed improvements for the system.* Indeed, CRDAMC had not only already begun to correct them, but had incorporated the strategic goals into mandatory yearly training and even published them in the medical literature.^16^ The absence of a strategic plan could have permitted the crisis to come and go without achieving substantial reform.

## Conclusions

Winston Churchill is credited with first saying “Never let a good crisis go to waste.” American political figure, Rahm Emanuel, riffed on the sentiment in 2008 when he stated “[a crisis] is an opportunity to do things you could not do before.” CRDAMC’s experience in the COVID-19 pandemic affirms that paradigm-changing growth is as available in a health-care crisis as it is in any other, if conditions are set to achieve it.
